# Editorial: Parenting in the Context of Opioid Use: Mechanisms, Prevention Solutions, and Policy Implications

**DOI:** 10.3389/fpsyg.2022.859257

**Published:** 2022-03-02

**Authors:** Leslie D. Leve, Elisabeth Conradt, Emily E. Tanner-Smith

**Affiliations:** ^1^Counseling Psychology and Human Services Department, Prevention Science Institute, University of Oregon, Eugene, OR, United States; ^2^Departments of Psychology, Pediatrics, and OB/GYN, University of Utah, Salt Lake City, UT, United States

**Keywords:** parenting, families, children, prevention, opioid use

The United States (U.S.) is experiencing an opioid epidemic of historic significance. In 2019, 10.1 million people misused prescription opioids in the past year (Substance Abuse Mental Health Services Administration, 2020) and more than 70,000 overdose deaths occurred (Hedegaard et al., 2020). The U.S. economic costs of this epidemic during 2017 alone were estimated at more than 1 billion USD (Florence et al., 2021). National-level epidemiological data from the U.S. indicate that the rates of opioid misuse, addiction, overdose, and fatalities are increasing at a particularly fast rate among women and individuals of childbearing and child-rearing age (Center for Disease Control Prevention, 2017; Hedegaard et al., 2020). Opioid-using behaviors among individuals who are parenting can have detrimental effects on their parenting and parent-child relationships, and can have downstream effects on child brain development, health, and subsequent risk for drug use (Wilens et al., 2002; Lander et al., 2013). Further, despite knowledge rooted in neuroscience that drug addiction is a disease, substance use in pregnancy is often perceived to be a choice, and women are frequently blamed for not having enough self-control to stop using substances (Schiff et al., 2021). Pregnant women who use opioids report having their pregnancy criminalized—experiencing harsh judgement by hospital staff (e.g., being called “drug addicts” or “dope fiends”) and/or being rejected from OB/GYN clinics because they are on medication assisted treatment (Syvertsen et al., 2021). These experiences threaten the likelihood that women seek and have access to prenatal care, access treatment for opioid use disorder, and access early intervention services for their children (Peacock-Chambers et al., 2020).

In 2017, the U.S. Department of Health and Human Services designated the opioid epidemic as a public health emergency and announced a 5-point strategy to combat this crisis, which included supporting cutting-edge research on addiction and improving access to prevention, treatment, and recovery support services (U.S. Department of Health Human Services, 2017). However, despite knowledge about the harmful associations between opioid use and parent and child development outcomes and national efforts to combat this crisis, significant gaps in the extant literature remain. Further, the COVID-19 pandemic has precipitated additional parenting challenges and stressors (Roos et al., 2021). Deaths related to opioid use are considered “deaths of despair” that have only magnified during this pandemic (Volkow, 2020). Given the well-established effects of substance use on parenting skills (Suchman et al., 2011, 2017; Lander et al., 2013) and the known effects of parental opioid use on infant and child development (Kocherlakota, 2014), addressing these knowledge gaps could improve the health and well-being of millions of children and families worldwide.

This book brings together 13 papers focused on increasing the scientific understanding of associations between parenting and opioid use. The papers cut across three broad themes: (1) theoretical and methodological insights that attend to the complex systems in which neurobiological, psychological, social, and structural features interact; (2) development and testing of new prevention and intervention programs; and (3) attention to a diversity of parent types and family structures. Cutting across all themes readers will notice attention to COVID-19 pandemic-related needs (see for example Clark et al.; DiPietro et al.; Smith et al.) and solutions (see Stormshak et al. for a telehealth example), and to the experiences of stigma (see Boeri et al.).

## Theme 1: Theoretical and Methodological Insights That Attend to the Complex Systems in Which Neurobiological, Psychological, Social, and Structural Features Interact

A common theme throughout this book is that identifying effective intervention targets for addressing opioid misuse in families requires attending to the complex systems in which neurobiological, psychological, social, and structural features interact. For instance, as documented in Barrett et al., Reese et al., and Swain and Ho, stress-sensitivity and reward dysregulation theories highlight the promise of developing interventions to target the neurobiological impacts of substance use on the brain and subsequent parenting behaviors. Nichols et al. illustrate how social ecological and systems theories offer promise for understanding how social contexts can serve as barriers or facilitators to opioid use prevention, treatment, and recovery efforts. Indeed, Smith et al. show how using causal loop diagrams as part of a systems science approach (e.g., Cruden et al.) can help document the complex pathways between individual and structural risk and protective factors, identifying mechanisms of change that may be promising levers for intervention. For instance, fear of stigmatization, concerns about surveillance from Child Protective Services, substance use treatment facility characteristics (e.g., hours of operation, costs, location, transportation, and childcare availability), and neighborhood built environment may represent significant barriers worthy of intervention, as highlighted in Boeri et al., Clark et al., and DiPietro et al. Multi-pronged intervention approaches that attend to upstream social determinants of health may thus offer unique promise for addressing opioid misuse in families.

## Theme 2: Development and Testing of New Prevention and Intervention Programs

This book also highlights the critical need for the development of evidence-based prevention and intervention programs designed specifically for families and parents when there is a history of opioid misuse. To our knowledge, the programs presented in this book are among the very first to be tested specifically for this population. Saldana et al. describe the results from a randomized trial of the Families Actively Improving Relationships (FAIR) program. Compared to those receiving traditional treatment services, parents in the FAIR program showed statistically and clinically significant improvements in parental opioid and methamphetamine use, mental health symptoms, parenting risk, and parenting stability. Six other reports describe promising new directions achieved through adaptations of existing interventions to better serve parents with a history of opioid misuse. Labella et al. describe an adaptation of the Attachment and Biobehavioral Catch-up intervention and provide case examples that highlight the challenges in working with this population as well as gains made by mothers. Cioffi and DeGarmo present an adaptation of Fathering through Change as an example of tailoring and accelerating the pace of science for this population. Stormshak et al. describe modifications to the Family Check-Up intervention for this population to allow for more wide-scale dissemination, ease of training with community providers, and increased public health reach for families in remote, rural areas. Barrett et al. present a video feedback intervention, Filming Interactions to Nurture Development, that can serve as a mechanistic experiment to illuminate mechanisms of change in interventions for this population. Cruden et al. describe the rigorous adaptation of the FAIR program (see Saldana et al.) to design a prevention-oriented intervention (PRE-FAIR). Finally, Reese et al. include a discussion of Mindfulness-Oriented Recovery Enhancement as a means of addressing mechanisms undergirding perinatal opioid use, parenting, and attachment. We are hopeful that this constellation of new and adapted prevention and intervention models, if ultimately shown to be effective, will increase the reach of services for families with a history of opioid misuse.

## Theme 3: Attention to a Diversity of Parent Types and Family Structures

Collectively, the articles in this book highlight the need for a diversity of parent types and family structures in studies of parenting in the context of substance use. For instance, Cioffi and DeGarmo describe how existing parenting interventions, largely evaluated with mothers, could be adapted to include fathers and other caregivers. Parents who were formerly incarcerated or who are involved in the child welfare system and are reentering both their communities and the parenting space are a uniquely vulnerable population in need of support. Clark et al. describe the parenting and other service needs as well as intervention recommendations for this population after surveying community service providers. Cruden et al. shed light on the need to adapt existing parent interventions to support prevention of initiation and escalation of opioid use for parents involved in child welfare. Nichols et al. focus on adolescents who may be at risk for opioid misuse, given their history of a substance use disorder. In order to have a broad reach, geographic diversity is also important. Stormshak et al. focus specifically on rural families, whereas Boeri et al. focus on barriers and motivators to opioid treatment in suburban areas. Finally, several contributions in this book focus on prenatal opioid use (Labella et al., Reese et al., DiPietro et al., Boeri et al.), in addition to this book's focus on postnatal use.

## Future Directions

This book collection highlights several important directions for future research and clinical work on parenting and opioid use. First, not only are more rigorous evaluations needed of prevention and intervention programs designed for families and parents with histories of opioid misuse, but cost analyses of these programs are needed to support efficient program planning with limited resources (see Saldana et al. for one example of this approach). Ideally, such efforts will be based in community-based participatory research methods to help strengthen the acceptability, viability, and effectiveness of interventions for this population. Second, there is a clear need for consideration of how problematic opioid use is defined and operationalized in the research literature, as the diverse definitions used by researchers can present challenges to synthesizing findings across the literature. Third, few studies have focused on both prenatal and postnatal periods, which makes it challenging to assess the causal pathways leading to child outcomes. Fourth, there is a critical need for more research and clinical work focused on parenting and opioid use in families of color. Prescription opioid use is highest among non-Hispanic whites and American Indian and Alaskan Natives, though recent reports suggest that opioid use is increasing in non-Hispanic Black adults (Harrison et al., 2018). Typically underreported are the rates of drug overdose deaths, which, in 2015, were highest among American Indian and Alaskan Natives (Mack et al., 2017). Increased representation of families of color is of utmost importance in future studies of opioid use in families.

## Author Contributions

LL, EC, and ET-S contributed to the conceptualization, writing, and editing of this editorial. All authors contributed to the article and approved the submitted version.

## Author Disclaimer

The content is solely the responsibility of the authors and does not necessarily represent the official views of the National Institutes of Health.

## Conflict of Interest

The authors declare that the research was conducted in the absence of any commercial or financial relationships that could be construed as a potential conflict of interest.

## Publisher's Note

All claims expressed in this article are solely those of the authors and do not necessarily represent those of their affiliated organizations, or those of the publisher, the editors and the reviewers. Any product that may be evaluated in this article, or claim that may be made by its manufacturer, is not guaranteed or endorsed by the publisher.
